# Immunohistochemistry defined subtypes of breast cancer in 678 Sudanese and Eritrean women; hospitals based case series

**DOI:** 10.1186/s12885-017-3805-4

**Published:** 2017-12-01

**Authors:** Asmerom Tesfamariam Sengal, Nada Suliman Haj-Mukhtar, Ahmed Mohammed Elhaj, Shahinaz Bedri, Eva Johanna Kantelhardt, Ahmed A. Mohamedani

**Affiliations:** 1 Pathology Department, Faculty of Medicine, University of Gezira, Wad-Medani, Gezira Sudan; 2 Orotta School of Medicine and Dentistry, Asmara, Eritrea; 3 National Cancer Institute, University of Gezira, Wad-Medani, Gezira Sudan; 4 Weill Cornell of Medicine- Qatar, Pathology and Laboratory Medicine, Department of Medical Education, Doha, Qatar; 5 Department of Gynaecology, Institute of Medical Epidemiology, Biostatistics and Informatcs, Martin-Luther University, (Saale) Halle-Wittenberg, Germany

**Keywords:** Hormone receptor, Breast cancer subtype, Immunohistochemistry, Sudan, Eritrea and Africa

## Abstract

**Background:**

Breast cancer is the most common malignancy accounting for 25% of all cancers in females. In Africa, breast cancer prevalence and mortality are steadily increasing. Knowledge of hormone receptors and human epidermal growth factor receptor-2 (HER-2) expressions are vital for breast cancer management plans and decision making. There is wide regional variation in the proportion of these biomarkers, especially in African countries. Hormone receptors positivity in indigenous African and African American women is considered to be low and triple negative breast cancer is a dominant phenotype. There is paucity of data regarding hormone receptors (ER and PR) and HER2 expressions in North-eastern Africa (Eritrea and Sudan). The purpose of this study was to evaluate the expression of ER, PR and HER2 in Eritrean and Sudanese case series and correlate these biomarkers with the clinicopathological profile.

**Method:**

Clinicopathologic data of patients were collected from clinical records. Immunohistochemistry biomarkers (ER, PR, and HER2) were assessed in consecutive female patients who had been diagnosed with invasive breast cancer from 2011 to 2015 in Gezira University Pathology Laboratory, the Sudan and National Health laboratory, Asmara, Eritrea.

**Results:**

There were 678 cases involved in this study. The mean age was 48.8 years with ±0.53 standard error of the mean. Two-thirds of the case were ≤50 years. Invasive ductal carcinoma, no special type was the most dominant histologic type (86%) in both study groups. The majority of cases (70%) had tumour stage pT2 and pT3 and about 50% had lymph node involvement. Less than 5% of the cases had well-differentiated tumours. The ER, PR and HER2 positive rates were 45%, 32%, and 29%, respectively. The proportion of luminal-A like, luminal-B like, HER2 enriched and TNBC were 37%, 13%, 16% and 34%, respectively. Fisher extract analysis showed age (*p* = .015), tumour size (*p* = .041), and histologic grade (*p* = .000) were significantly associated with intrinsic subtypes. Furthermore, Logistic regression analysis stratified by origin, age, tumour size, lymph-node metastasis and grade indicated that younger women age (≤50 years) and grade III tumours were more likely to be diagnosed with ER negative breast cancer.

**Conclusion:**

Most of Sudanese and Eritrean women were diagnosed at younger age and with unfavourable prognostic clinicopathologic prognostic markers. TNBC is more frequent in this cohort study; patients with grade III tumours and young age are more likely to be hormone receptors negative. Therefore, routine determination of hormone receptors is warranted for appropriate targeted therapy.

## Background

Breast cancer (BC) is the most prevalent cancer in women accounting for one fourth of all cancers and is the second cause of cancer-related death in both developed and most developing countries [1–3]. In Africa, the incidence of BC is relatively low compared to the western developed countries however, mortality rates are alarmingly high. Epidemiological data from Surveillance, Epidemiology, and End Results (SEER) documented a gradual drop in BC incidence and mortality in white American but a steady increase in black American women [4]. BC is a heterogeneous disease both in clinical and pathological profiles. There is a wide variation in clinical presentation, histologic type, molecular biomarkers, prognosis, and treatment outcome.

BC in African and African American (AA) women has been reported to be a more aggressive disease [5, 6]. Many studies showed women with BC in native African, African in diaspora and AA had been diagnosed at a younger age, with higher histologic grade, advanced stage and with higher hormone receptor (HR) negative proportions than white women [5, 7–10]. A recent, investigation revealed unique genetic polymorphisms that are associated with hormone receptor negative BC in African American women.

Hormone receptors (oestrogen receptor (ER) and progesterone receptor (PR)) and human epidermal growth factor receptor-2 (HER2) are the most relevant clinical biomarkers that are widely used in stratifying BC cases management. The rate of ER, PR, and HER2 of BC varies from region to region. Patients with HR positivity have a better prognosis and are eligible for hormonal targeted therapy. The prevalence of these biomarkers is inconsistent and there is wide variation among ethnicities. A meta-analysis review in indigenous African women reported a wide range of ER positive BC (40 to 80% in North Africa and 20 to70% in West Africa) [11]. It is also reported AA women with BC had less ER/PR positive tumours compared to Caucasian women. In general, African women have higher HR negative BC tumours [12] compared to other races although a recent data reported an increasing trend in some African countries [13, 14].

Perou and colleagues clustered breast cancer based on DNA microarray signature into luminal-A, luminal B, HER2 enriched, basal like and normal like [15]. Following to this investigation, many studies classified BC molecular subtypes using IHC surrogate markers in a similar way to the DNA microarray clustering [16]. The luminal A (ER+ and/or PR+ and HER2 -) is more common in older Caucasian women and has superior prognosis and treatment outcome. Luminal B (ER+, PR+ and HER2+) is also common in white women and has a worse prognosis compared to luminal-A, but better than the HER2 enriched and basal like [17]. HER2 enriched is more aggressive and common in younger women but with the introduction of targeted therapy anti- HER2 (Herceptin, monoclonal antibody) the outcome of BC patients expressing HER2 slightly improved. The basal like is 80% concordant to the triple negative BC (TNBC); it is more common in indigenous African women and young African American women and has poorest prognosis and treatment outcome [18, 19].

Despite the growing descriptive studies which are mostly based on IHC markers of HR and HER2 expression evaluation, the detailed genetic landscape of breast cancer in indigenous African women is poorly investigated.

The distribution of the receptor defined BC subtypes in Africa is highly variable like the ER and PR rate. Although a number of studies reported the TNBC is most common molecular subtype in Africa [20] a recent study reported breast cancer in West African women is different from Eastern African women. For instance, one study documented Ethiopian women with breast cancer have comparable percentage of luminal-A subtype to those white Americans [5]. On the other hand, Western African women from Ghana have more TNBC as compared to Ethiopian and White American women [5]. There is also regional difference in the proportion of subtype of BC within one country. Awadelkerim et al. reported a high rate of HR positive subtype which is comparable to white Caucasian women (Italian) [21]. Our cohort study and a recent report from Khartoum found HR negative BCs are more prevalent in Sudanese women [22]. There is a huge disparity in HR status and molecular subtypes of breast cancer among nations and within a nation. What contributes to regional and racial differences in the proportion of molecular subtypes of BC is not clear. It is crucial to know the distribution of ER/PR and HER2 expression and their derived BC subtypes in outlining strategic BC management plans in this region. Due to limited resources, ER/PR and HER2 determination are not done routinely in clinical practice in Eritrea and Sudan. The purpose of this study was to investigate the expression of ER, PR, HER2 and receptor defined molecular subtypes in north-east African women (Sudanese and Eritrean) and analyse the correlation of clinical and histologic markers with HR status and molecular subtypes.

## Methods

### Data collection

Ethical approval was obtained from the research ethics committees of both institutions (University of Gezira and Orotta School of Medicine and Dentistry). Clinical data were retrieved using a standard protocol from the histopathology department of the University of Gezira (UOG), Gezira, Sudan and Orotta School of Medicine and Dentistry (OSMD) in National health laboratory, Asmara, Eritrea. This study was a retrospective facility based consecutive case series from 2011 to 2015 with histologically confirmed invasive breast carcinoma and not treated previously. Demographic data and tumour characteristics were obtained from medical records and hormone receptor status ER, PR and HER2 expressions were determined using standard IHC method. Tumour grade was assessed according to the modified Nottingham Bloom-Richardson grading system [23]. Tumour size and nodal status were described according to the TNM classification [24].

### Study setting

Eritrea and Sudan are located in the Northeast of Africa. Eritrea has six million people and has only one national Anatomic-pathology department where all clinical histology and cytology are assessed. Sudan is the largest country in Africa with more than 40 million people. Sudan has diverse ethnic groups including Afro, Arab and Afro-Arab tribes. Gezira state has about four million inhabitants; the University of Gezira histopathology laboratory is the only public institution serving this state and other nearby states. There is no radio/chemotherapy centre in Eritrea. But Sudan has two public oncology hospitals located in Khartoum and Wad-Medani (Gezira). The national cancer institute of Gezira state was established in 1999 and serves for almost half of the country including some referral cases from Eritrea.

### Immunohistochemistry

For both Sudanese and Eritrean cohort case series, ER, PR, and HER2 immunostaining was performed manually in University of Gezira (UG), pathology laboratory, Wad-Medani, Sudan and National health laboratory (NHL) pathology department, Asmara, Eritrea, respectively. In brief, formalin-fixed paraffin-embedded (FFPE) breast tumour blocks were obtained from pathology department of UG and NHL. FFPE tissues were sectioned serially into 4 μm and placed in frosted microscopic slides and deparaffinized in series of xylene (three changes), graded alcohol (2 changes 100%, 90%, and 70% ethanol) and rehydrated in distilled water. Antigen retrieval was performed using a water bath in 10 mM citrate buffer (pH 6.0) at 95 °C for 45 min. Then washed with Tris Buffered Saline and blocked with 3% hydrogen peroxide in Phosphate Buffered Saline. After that tissue sections were blocked with background snipper using a blocking agent (Biogenex, UK). Then incubated for 1 h with primary antibodies at room temperature: anti-ER (clone EPR703, Biogenex UK), anti-PR (clone PR88, Biogenex Ltd., UK), and anti-HER2neu (clone CB11), followed by biotinylated horse anti-mouse or goat anti-rabbit secondary antibodies. Staining was visualized using Diaminobenzadine (DAB) and counterstained with haematoxylin.

ER and PR were considered positive if ≥1% nuclei of tumour cells were stained as per the American Society College Oncology/College American Pathology (ASCO/CAP) guidelines [25] for both Sudanese and Eritrean women. HER2 was scored as 0, 1+, 2+, or 3+ and FISH was not done for equivocal (2+) HER2 results in both groups and only with score 3+ was considered HER2 positive and ≤2+ score was assumed HER2 negative. Molecular breast cancer subtypes were defined using combination of these IHC markers as follows: luminal A-like (ER positive and/or PR positive and HER2 negative), luminal B-like (ER positive and/or PR positive/PR negative and HER2 positive), HER2 enriched type (ER negative, PR negative, HER2 positive), and triple negative (ER, PR, and HER2 negative) [13, 26, 27].

### Data analysis

Data analysis was performed using SPSS version 21. Tumour characteristics and biomarkers of Sudanese and Eritrean women were compared across the BC subtypes using the chi-squared (X^2^) test for categorical variables. Logistic regression analysis was used to determine the odd ratio (OR) or relative risk to evaluate the effect of age, histologic type, tumour size, lymph node metastasis and Nottingham histologic grade on a probability of ER-negative tumours or tumour subtypes. All *p*-values were calculated based on two-tailed tests of significance, where *p* < 0.05 was considered statistically significant.

## Results

**Fig. 1:**
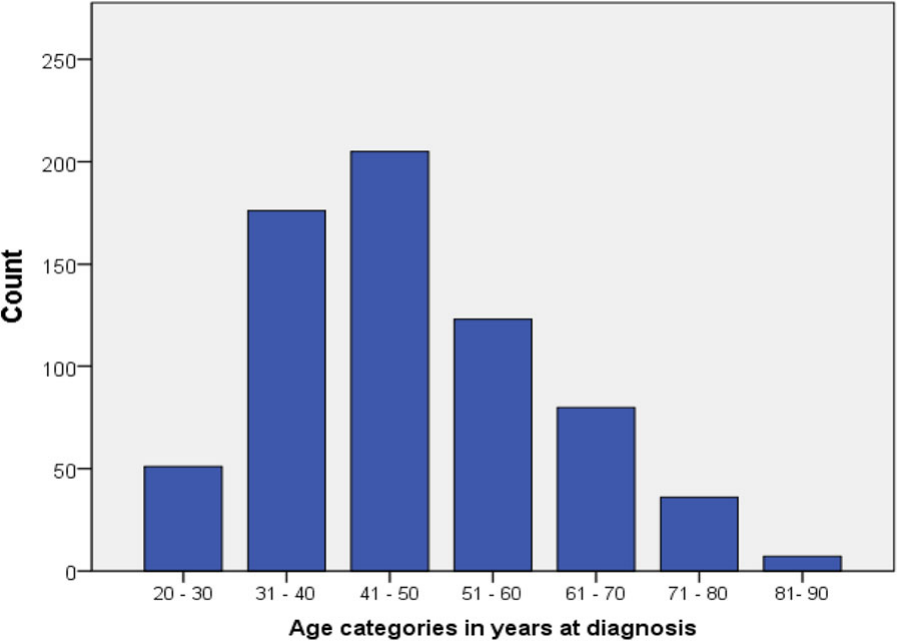
Distribution of Breast cancer in both Eritrean and Sudanese women according to age category

A total of 678 patients (116 Eritrean and 562 Sudanese women) were included in this study. The age ranged from 20 to 90 with a mean age at diagnosis 48.8 years with the standard error of the mean (SEM) ±0.53. Two-thirds of patients were under 50 years and age category distribution is presented in (Fig. 1). The peak age category was 40–51 years.

The proportion of tumour characteristics, HR status and IHC defined molecular subtypes of both groups are summarized in Table 1.

**Table 1:** proportion of clinicopathologic and IHC defined subtypes of BC in Eritrean and Sudanese women

Clinical and tumour characteristics		Origin				Combined both study groups	
		Eritrean		Sudanese			
		*n*	%	*n*	%	*n*	%
Histologic type	IDCNST	89	76.7%	492	87.9%	581	85.9%
	ILC	11	9.5%	18	3.2%	29	4.3%
	others	16	13.8%	50	8.9%	66	9.8%
Pathologic tumour size	pT1	23	19.8%	82	15.5%	105	16.3%
	pT2	48	41.4%	248	47.0%	296	46.0%
	pT3	37	31.9%	118	22.3%	155	24.1%
	pT4	8	6.9%	80	15.2%	88	13.7%
LN involvement	pN0	72	62.1%	198	47.3%	270	50.5%
	pN1(1–3)	15	12.9%	86	20.5%	101	18.9%
	pN2(3–9)	16	13.8%	78	18.6%	94	17.6%
	pN3(>9)	13	11.2%	57	13.6%	70	13.1%
Grading	GI	10	8.6%	20	3.6%	30	4.4%
	GII	46	39.7%	306	54.6%	352	52.1%
	GIII	60	51.7%	234	41.8%	294	43.5%
LVI	NO	44	37.9%	264	46.9%	308	45.2%
	unknown	6	5.1%	111	19.8%	117	17.3%
	YES	56	48.3%	185	15.2%	102	35.5%
ER	ER negative.	59	50.9%	308	55.0%	367	54.3%
	ER positive.	57	49.1%	252	45.0%	309	45.7%
PR	PR negative	69	59.5%	346	61.8%	415	61.4%
	PR positive	47	40.5%	214	38.2%	261	38.6%
HR	HR positive	57	49.1%	257	45.9%	314	46.4%
	HR negative	59	50.9%	303	54.1%	362	53.6%
HER2	negative	82	70.7%	296	71.3%	378	71.2%
	positive	34	29.3%	119	28.7%	153	28.8%
	Missing	0	0	147	–	147	–
IHC based breast cancer subtype	Luminal A-like	43	37.1%	153	36.9%	196	36.9%
	Luminal B-like	14	12.1%	54	13.0%	68	12.8%
	HER2 enriched	20	17.2%	65	15.7%	85	16.0%
	TNBC	39	33.6%	143	34.5%	182	34.3%

*IDCNST* invasive ductal carcinoma, no special type, *ILC* invasive lobular carcinoma, *TNBC* Triple negative Breast cancer

The most common morphologic type of breast cancer in this study cohort was invasive ductal carcinoma no special type (NST). Invasive lobular carcinomas account for less 5% when combining both study groups (3.2% in Sudanese and 10% in Eritrean women). About16% had tumour size less than 2 cm (pT1) and about one third had tumour size greater than 5 cm (pT3and pT4). Half of the patients had lymph node metastasis and only 35.5% had lymphovascular invasion (LVI). About 96% of patients were diagnosed with moderate to poorly differentiated (grade II/III) tumours. Of note, half of the Eritrean and 44% of Sudanese women presented with grade III (Table 1).

In regard to HR status, 54% women with BC were negative for ER and/or PR. The proportions in ER negative were similar in both study group; 51% and 55% for Eritrean and Sudanese women, respectively. For grade I cases, 75% (24/32) were ER positive. Similarly, (25/28) of ILC were positive for ER merging both groups (Table 2).

**Table 2:** ER and PR proportion according to tumour characteristic in both Eritrean and Sudanese women

Tumour and biomarker characteristics		Eritrean				Sudanese			
		ER positive		ER negative		ER positive		ER negative	
		*n*	(%)	*n*	(%)	*n*	(%)	*n*	(%)
Histologic type	IDCNST	41	71.9%	48	81.4%	222	83.6%	270	91.5%
	ILC	10	17.5%	1	1.7%	15	5.7%	3	1%
	Others	6	10.5%	10	16.9%	27	10.7%	23	7.5%
Pathologic Tumour size	pT1	17	29.8%	6	10.2%	46	19.2%	36	12.5%
	pT2	26	45.6%	22	37.3%	107	44.6%	141	49.0%
	pT3	10	17.5%	27	45.8%	50	20.8%	68	23.6%
	pT4	4	7.0%	4	6.8%	37	15.4%	43	14.9%
Lymph node involvement	pN0	40	70.2%	32	54.2%	104	52.0%	94	42.9%
	pN1	6	10.5%	9	15.3%	37	18.5%	49	22.4%
	pN2	8	14.0%	8	13.6%	36	18.0%	42	19.2%
	pN3	3	5.3%	10	16.9%	23	11.5%	34	15.5%
Nottingham Grade	GI	8	14.0%	2	3.4%	16	6.3%	4	1.3%
	GII	30	52.6%	16	27.1%	155	61.5%	151	49.0%
	GIII	19	33.3%	41	69.5%	81	32.1%	153	49.7%
PR	PR negative	10	17.5%	59	100.0%	43	17.1%	303	98.4%
	PR positive	47	82.5%	0	0.0%	209	82.9%	5	1.6%
HER 2	Negative (0 and 1+)	37	64.9%	29	49.2%	132	65.1%	112	52.8%
	Negative (2+)	6	10.5%	10	16.9	20	9.8%	32	15.1%
	Positive (3+)	14	24.6%	20	33.9%	51	25.1%	68	32.1%

*IDCNST* invasive ductal carcinoma, no special type, *ILC* invasive lobular carcinoma

**Fig. 2:**
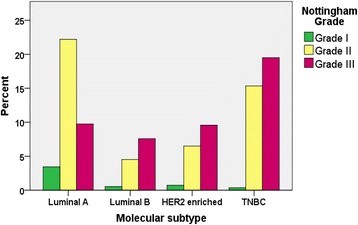
Distribution of IHC defined intrinsic subtypes of BC according to grade in both Eritrean and Sudanese women

HER2 negative rate was the same in both Eritrean and Sudanese women (71%). In 147 Sudanese cases, HER2 was not determined due to inadequate tissue for assessment. Data analysis was made removing these cases to determine the proportion positive, negative and equivocal results for HER2 in Table 2 and the same approach was used for subtyping. Interestingly, the percentage of molecular subtypes of BC in both countries was quite same (Tables 1 and 3). Nearly one-third (37%) had luminal-A like and 34% of women were TNBC, which have superior and poor prognosis, respectively. The majority of TNBC were grade II or III and larger tumour size, while patients with luminal-A like had well to moderately differentiated tumours and smaller tumour size (Fig. 2 and Table 3).

**Table 3:** Association of demographic and clinicopathologic profiles with IHC defined subtypes of BC in both Eritrean and Sudanese women

Demographic and tumour characteristics		IHC defined breast cancer subtypes								*P* value
		Luminal A		Luminal B		HER2 enriched		TNBC		
		n	%	n	%	n	%	n	%	
Age in years	≤50	131	37.1%	35	9.9%	53	15.0%	134	38.0%	0.015
	>50	65	32.3%	35	17.4%	40	19.9%	61	30.3%	
Nationality	Sudanese	153	35.0%	56	12.8%	73	16.7%	155	35.5%	0.924
	Eritrean	42	36.2%	14	12.1%	20	17.2%	40	34.5%	
Histologic type	^a^IDCNST	159	33.3%	65	13.6%	85	17.8%	168	35.2%	0.056
	^b^ILC	16	61.5%	1	3.8%	5	19.2%	4	15.4%	
	Medullary	0	0.0%	0	0.0%	0	0.0%	13	100.0%	
	Mucinous	16	94.1%	1	5.9%	0	0.0%	0	0.0%	
	Papillary	1	6.7%	3	20.0%	2	13.3%	9	60.0%	
	Cribriform	4	100.0%	0	0.0%	0	0.0%	0	0.0%	
Pathologic tumour size	pT1	45	47.9%	14	14.9%	10	10.6%	25	26.6%	.041
	pT2	74	31.1%	35	14.7%	48	20.2%	81	34.0%	
	pT3	43	31.2%	13	9.4%	23	16.7%	59	42.8%	
	pT4	27	40.3%	7	10.4%	9	13.4%	24	35.8%	
Lymph Node involvement	pN0	102	42.7%	31	13.0%	34	14.2%	72	30.1%	0.58
	pN1	22	27.5%	10	12.5%	19	23.8%	29	36.3%	
	pN2	25	29.8%	15	17.9%	14	16.7%	30	35.7%	
	pN3	12	21.4%	8	14.3%	7	12.5%	29	51.8%	
Nottingham Grade	GI	19	67.9%	3	10.7%	4	14.3%	2	7.1%	0.000
	GII	123	45.7%	25	9.3%	36	13.4%	85	31.6%	
	GIII	54	21.0%	42	16.3%	53	20.6%	108	42.0%	

^a^ *IDCNST*= invasive ductal carcinoma, no special type, ^b^ *ILC*= invasive lobular carcinoma

There was significant association between age (*p* = 0.015), pathologic tumour size (*P* = 0.041) and histologic grade (*p* = .000) with the BC subtypes but no association was found with nationality/origin (*p* = 0.924), histologic type (*p* = 0.056), and lymph node involvement (*p* = 0.058) as show in (Table 3). We noted some special type like medullary and papillary histologic types were TNBCs and histologic types of lobular and mucinous carcinomas were HR positive (data not shown).

Furthermore, multivariate logistic regression analysis stratified by age, origin, tumour size, lymph node status and grade also revealed no association between origin, histologic type and lymph node involvement with ER negativity. On the other hand, younger age (≤50 years) (*P* = 0.039, OR = 1.50, 95 CI 1.02–2.15), pT3 and Nottingham grade III were significantly associated with ER negative BC (*p* = .000, OR 2.2, 95% CI 1.5–3.2) (Table 4).

**Table 4:** Multivariate logistic regression analysis for relative risk ratio of ER negative BC stratified by age, origin, pathologic tumour size, lymph-node metastasis and grade in both groups of cohort study (Eritrean and Sudanese women)

Clinical and tumour characteristics		*p*-value	Risk ratio	95% Confidence interval	
				Lower	Upper
Age in years	Age ≤ 50 compared to >50	.039	1.480	1.019	2.149
Origin	Eritrean (reference Sudanese)	.956	.988	.634	1.537
Histologic type	^a^IDCNST (reference)	.198			
	Invasive lobular carcinoma	.051	.847	.780	1.211
	other	.532	.833	.469	1.479
Pathologic Tumour size	pT1 (reference)	.081			
	pT2	.154	1.438	.873	2.368
	pT3	.013	2.050	1.162	3.615
	pT4	.632	1.194	.577	2.471
Lymph Node involvement	pN0 (reference)	.306			
	pN1	.149	1.432	.880	2.332
	pN2	.694	1.106	.670	1.823
	pN3	.133	1.556	.875	2.768
Grade	GIII (GI/II reference)	.000	2.203	1.534	3.164

^a^ *IDCNST*= invasive ductal carcinoma, no special type

## Discussion

Our cohort study is presenting a large case series data from northeast Sub-Saharan Africa (Eritrea and Sudan) countries. This region is a disadvantaged region with inadequate oncology service. There are no cancer screening programs and prevention strategies aiming in cancer reduction in this region [28]. BC account for more than 25% of all cancers in Eritrea [29] and an equal figure of deaths was estimated by WHO in this region [30]. This study indicated that majority of women in this region with BC were diagnosed at younger age (≤50 years) with mean age 48.8 years consistent with previous reports in Africa [31]. Invasive ductal carcinoma (NST) was the most dominant histologic type, and nearly half of patients had lymph node involvement. ILC often express HR and has good prognosis is less frequent and especially in Sudanese women. Our study revealed less than 5% of women with BC have well-differentiated tumours and the majority had T2 or T3 tumour stage. Similar findings have been reported from Khartoum [21] and in native African women [31, 32]. Generally, aggressive clinical markers of BC are a common phenomenon in most developing countries. This could be partially due lack of screening and late diagnosis or some intrinsic biologic factors that have not yet been addressed.

Knowledge of hormone receptor and molecular subtype in a certain population is crucial in implementing breast cancer treatment plans. We found more than half (54%) of women with BC had ER negative and 62% PR negative. Our result is remarkably lower compared with the finding from West African women [33–35], Tunisia [36] Uganda [37] but much higher as compared with those reported from Egypt [38], Ethiopia [39], South African women [13] and Caucasian women in the west. Our finding is also different from previously reported in Central Sudan (Khartoum) [21] but similar to a recent report by Eltaib et al. [22]. The former study was small sample size, with a possible intrinsic bias and might not represent the actual population.

Multivariate logistic regression analysis in our study showed younger women (age ≤ 50 years) are more likely to develop ER negative BC (*P* = 0.039, OR = 1.50, 95% CI 1.02–2.15). Similarly, grade III tumours are two times higher than grade I or II (*p* = .000, OR 2.2, 95% CI 1.5–3.2). This finding is consistent with previous studies that younger premenopausal women (<50 Years) and poorly differentiated BC is more likely to be HR negative [40]. The triad of poor clinical markers (young age, HR negative and poorly differentiated tumour) are common features in many African women with BC.

Another remarkable finding in this study is higher proportion of TNBC which accounted for 34% in both study groups. Our result is in agreement with many reports from African studies and other developing countries [41]. This finding is almost the same with BC in black premenopausal women from Carolina breast cancer study (CBC) [40] and a recent report of the cancer Genomic Atlas (TCGA) data analysis in AA women (33%) [42]. But our finding is significantly lower compared with the white American women in the aforementioned studies (34% versus15%) [42]. The TNBC is both clinically and biologically distinct disease. It is more common in younger women, poorly differentiated, invasive ductal carcinoma, no special type which are all present in our case series of TNBCs. Patients with TNBC are not responsive to standard chemotherapy and have a poor outcome. Women with TNBC have lower disease free survival and overall survival in many African American studies. It is also reported that BRCA1/2 mutations are more common in TNBC. TCGA data analysis has reported higher TP53 and MLL3 mutations in black African American than white American women with TNBC but no difference in somatic copy number of mutations [42]. Another study also reported amplification of fibroblast growth factor receptor 2 (FGFR2) genes in TNBC but not in other subtypes. However, the genetic profile of TNBC in indigenous African women is not fully investigated.

The expression of HER2 in this cohort study was remarkably high (28%); a similar rate have been reported in some African studies and Saudi Arabian women [43] and south Asian [44, 45]. Luminal-A like subtype which is less aggressive type of BC was less frequent (37%) in our study population but the proportion is consistent with AA of CBC study [40]. This figure is lower compared to Egypt [38], Morocco [31] some other East African countries but higher compared to West African studies (Nigeria and Senegal) [33]. Previous data from Khartoum reported a higher proportion of luminal A which is comparable to Caucasian women with BC [21], but patients recruited in this study was too small (only one fifth of our study).

It is of note that there is a wide variation in prevalence of HR status, HER2 and their derived intrinsic subtypes of BC among African women as well as between blacks and whites. Our finding does not fit either to extremely low rates reported in West Africa nor to the remarkably high rate in Eastern and North Africa but consistent with AA. The reason for this racial and regional disparity of subtypes of BC remained an open question. The potential contributors for this disparity could be technical (quality of tissue fixation, processing, various staining techniques, and different criteria for scoring and reporting). In our cohort, we used similar laboratory methods of evaluation and there is no significant difference in the prevalence of the biomarkers and their derived subtypes in the two countries. It is also possible that the molecular phenotypes of African BC are biologically diverse and possibly due to different genetic polymorphisms within or among population, as well as reproductive pattern and environmental factors. For example, the prevalence of BRCA1/2 mutations is not significantly higher in AA women as compared to Caucasian- American women, in spite of this finding that TNBCs are more frequent in AA [46], suggesting that there are probably other genetic pathways for this molecular subtype. The potential contribution of reproductive related factors is supported by the finding that TNBC in AA women tends to increase with increased parity and younger age at first full-term pregnancy.

There are some limitations to this study: the small sample size from Eritrean women case series, a retrospective clinical data collection and lack of evaluation for HER2+ equivocal results using fluorescent in-situ hybridization (FISH) and not performing Ki67 as a marker of proliferative index. We only considered HER2 positive when IHC score is 3+ and the actual HER2 positive could be more than the figure we have reported as some of the equivocal (HER2) 2+ might be positive by FISH. Furthermore, not all Sudanese patients were evaluated for HER2 this could affect our inferences.

## Conclusion

In conclusion, breast cancer in Eritrean and Sudanese women is more common in younger age and dominated by more aggressive clinical and molecular prognostic markers. Younger age and poorly differentiated (grade III) tumours are strongly associated with ER negative breast cancer. The luminal-A like which is indolent subtype and sensitive to hormone therapy is less frequent, instead, the most aggressive subtype (TNBCs) are more prevalent in our study group.

## Abbreviations

AA: African American
BC: Breast cancer
ER: Oestrogen Receptor
HER2: Human Epidermal growth factor Receptor 2
IDCNST: Invasive Ductal Carcinoma No Special Type
IHC: Immunohistochemistry
ILC: Invasive Lobular Carcinoma
PR: Progesterone Receptor
TNBC: Triple Negative Breast Cancer
